# Adiposity increases weight-bearing exercise-induced dyspnea despite favoring resting lung hyperinflation in COPD

**DOI:** 10.1177/14799731211052305

**Published:** 2022-02-05

**Authors:** Zewari S, van den Borst B, van den Elshout FJ, Vercoulen JH, Dekhuijzen PN, Heijdra YF, Vos PJ

**Affiliations:** 1 Department of Pulmonary Disease, 1321 Rijnstate Hospital, Arnhem, The Netherlands; 2 Department of Pulmonary Disease, 6034 Radboud University Medical Center, Nijmegen, The Netherlands; 3 Department of Medical Psychology and Department of Pulmonary Diseases, 6034 Radboud University Medical Center, Nijmegen, The Netherlands

**Keywords:** COPD, adiposity, obesity, dyspnea

## Abstract

**Objectives:**

Our aim was to study the associations between resting lung hyperinflation, weight-bearing exercise-induced dyspnea and adipose distribution in obese and normal-weight COPD patients.

**Methods:**

We performed a comparison between 80 obese COPD patients (COPD_OB_) with 80 age- and FEV_1_ matched normal-weight COPD patients (COPD_NW_). Dyspnea was assessed by the mMRC scale and the Borg dyspnea score before and after a 6 min walk test. Further characterization included spirometry, body plethysmography and metronome paced tachypnea (MPT) to estimate dynamic hyperinflation. Body composition was assessed with bioelectrical impedance analysis. Associations between dyspnea scores and BMI and body composition groups were studied using logistic regression models.

**Results:**

COPD_OB_ patients had attenuated increases in TLC, FRC and RV compared to COPD_NW_ patients (*p* < 0.01). The groups had comparable 6 min walking distance and ΔFRC upon MPT (*p* > 0.05). Compared to COPD_NW_, COPD_OB_ patients reported more often a mMRC ≥ 2 (65 vs 46%; *p* = 0.02; OR 3.0, 95% CI 1.4–6.2, *p* < 0.01) and had higher ΔBorg upon 6MWT: 2.0 (SEM 0.20) vs. 1.4 (SEM 0.16), *p* = 0.01; OR for ΔBorg ≥ 2: 2.4, 95% CI 1.1–5.2, *p* = 0.03. Additional logistic regression analyses on the associations between body composition and dyspnea indicated that increased body fat percentage, fat mass index and waist-to-hip ratio were associated with higher ORs for mMRC ≥ 2 and ΔBorg upon 6MWT ≥ 2.

**Conclusion:**

Despite its beneficial effect on resting lung hyperinflation, adiposity is associated with increased weight-bearing exercise-induced dyspnea in COPD.

## Introduction

Chronic Obstructive Pulmonary Disease (COPD) and obesity are major health problems and the prevalence of both disorders is increasing.^1,2^ Dyspnea, particularly exercise-induced, is one of the predominant and most disturbing symptoms in patients with COPD,^3^ and is considered an even more important risk factor for mortality than the degree of airflow limitation.^4^ Dyspnea has been defined as a subjective experience of breathing discomfort that consists of qualitatively distinct sensations that vary in intensity, and many physiological and psychological factors can have an influence on dyspnea.^5^

Most COPD patients have some degree of resting lung hyperinflation.^6^ Hyperinflation is one of the mechanisms leading to dyspnea in COPD by causing mechanical limitation to increase tidal volumes, increasing elastic recoil and affecting airway stretch receptors.^7^ Obesity in itself also affects dyspnea through various mechanisms.^8–10^ Focusing primarily on the pulmonary function, it is believed that pulmonary function in otherwise healthy obese subjects is affected by several factors including a mass effect of extra-thoracic adipose tissue and increased intra-abdominal pressure by local abdominal adiposity.^11–13^ This can result in excess bibasal airway collaps, increased small airway resistance, local airtrapping and diffuse microatelectasis. This, in turn will increase static lung elastic recoil pressure leading to lower end-expiratory lung volumes (EELV) and lower functional residual capacity (FRC).^9,12^ Collectively, the net result is an increase in the work of breathing being associated with increased sense of shortness of breath.^10,14^ There is a complex interaction between COPD and obesity in terms of effects on pulmonary function.^15,16^ When combined, it has been consistently observed that obese COPD patients tend to have attenuated increased resting lung hyperinflation compared to non-obese COPD patients, even when controlled for degree of airflow limitation.^17^

In the study of exercise-induced dyspnea in obese COPD, it is important to note that inconsistencies between studies appear to be related to a difference between weight-supported and weight-bearing exercise protocols. For example, studies examining lung function dynamics and dyspnea during weight-supported symptom-limited cycling tests have shown that obesity has no negative influence on dyspnea.^18–20^ In these studies dyspnea measured by Borg dyspnea scores were comparable between normal-weight and obese COPD patients. More conflicting results have been produced by studies assessing weight-bearing exercise-induced dyspnea in obese COPD patients. Some studies indicate that dyspnea levels, measured with different tools (Borg scores during 6MWT and/or mMRC), are comparable between obese and normal-weight COPD.^21–25^ However, there is also accumulating data reporting increased dyspnea in obese COPD patients.^22,26–28^

In a cross-sectional comparison between age- and FEV_1_-matched obese and normal-weight COPD patients, we investigated the association between resting lung hyperinflation and weight-bearing exercise-induced dyspnea and explored the effects of body fat distribution on these associations. We hypothesized that markers of adiposity are associated with increased weight-bearing exercise-induced dyspnea in COPD, independent of the degree of resting lung hyperinflation.

## Methods

### Subjects

We studied COPD patients, defined according to the GOLD definition,^2^ who were either obese (BMI ≥ 30 kg/m^2^) or normal-weight (BMI 18.5–25 kg/m^2^). There were no restrictions with regard to sex or the severity of airflow limitation. Subjects were clinically stable (i.e. no history of exacerbation in the previous 2 months) and were >18 years of age. Exclusion criteria were recent acute cardiovascular events, unstable cardiac arrhythmia, neuromuscular disorders and respiratory comorbidity other than COPD, such as asthma and lung diseases causing restriction (interstitial lung diseases) to minimize confounding. Furthermore, patients who were unable to perform pulmonary function tests (PFT), 6MWT or fill out the questionnaires were excluded.

### Study design

This study was performed at a teaching hospital in The Netherlands. The study was approved by the regional committee and local ethics committee (reference 1026/160614). Consecutive obese COPD patients (COPD_OB_) visiting the pulmonary outpatient clinic who met the inclusion criteria were asked to participate. For each COPD_OB_ participant, one age-matched (±5 years) and FEV_1_-matched (±5 %predicted; based on the most recent PFT) normal-weight COPD patient (COPD_NW_) was asked to participate. Of the 394 eligible COPD patients, a total of 160 were willing to participate. All subjects completed two visits to perform the tests. We obtained informed consent from all subjects.

### Procedures

During the first visit, the modified Medical Research Council (mMRC) dyspnea scale ^29^ and the Saint George Respiratory Questionnaire (SGRQ) ^30,31^ were assessed. Subjects were asked about their tobacco exposure and smoked pack years were calculated. During this visit anthropometric measurements (weight, height, waist circumference and hip circumference) were obtained. Body composition was measured with bioelectrical impedance analysis (Bodystat 1500; Bodystat, UK). Fat-free mass index (FFMI) was calculated as the ratio of FFM to height in meters squared. Known comorbidities were retrieved from hospital files.

During the second visit spirometry, body plethysmography and 6MWT were performed. The walk work during 6 MWT (6MWW) was calculated as a product of distance x body weight). The European Community for Coal and Steel reference equations were used to calculate predicted values.^32^ The Borg dyspnea score was assessed at rest and at the end of 6MWT. Furthermore, Metronome Paced Tachypnea (MPT) was performed for detection of dynamic hyperinflation. During the MPT test, a respiratory rate twice the baseline rate for 20s is achieved in patients, which is immediately followed by sequential measurement of inspiratory capacity.^33^ Trained staff performed these tests in accordance with American Thoracic Society/European Respiratory Society guidelines^34^ and were not aware of the study goals.

After completion of the PFT, three subjects in the COPD_OB_ were excluded because they had a TLC < 80% predicted. As a consequence, we also excluded their three matched COPD_NW_ peers for analyses. Consequently, 80 COPD_OB_ and 80 COPD_NW_ subjects were included in the analyses. Diffusion capacity could not be measured in two COPD_OB_ and two COPD_NW_ subjects (technical reasons); body box measurements could not be measured in one COPD_NW_ because of claustrophobia; 6MWT results were missing for two COPD_OB_ (not able to walk at the day of the test because of hip problem and fractured leg) and one COPD_NW_ (technical reason); dynamic hyperinflation results were missing in 1 COPD_OB_ (technical reason).

### Statistics

Descriptive statistics were used to characterize the study population. Continuous variables are expressed as mean (SD) and discrete variables are shown as percentages. Comparisons of means for continuous variables were conducted by using t-tests (two-tailed). Proportions of categorical variables were compared by Chi-squared test (two-tailed). Additional hypothesis-generating post hoc analysis was also performed to better understand the role of obesity and measures of body composition on dyspnea. Binary logistic regression models were used to study independent associations between COPD subgroups, body composition measures and dyspnea. A *p* value <0.05 was considered to be statistically significant. Analyses were performed using SPSS version 20.0 (IBM, USA).

## Results

Table 1 shows the comparisons of demographics, anthropometry, body composition, comorbidities and QoL between the groups. The groups were comparable in terms of sex distribution, pack years smoked and smoking status. None of the patients used long-term oxygen therapy. Although SGRQ scores tended to be higher in the COPD_OB_ patients, these differences were not significant.

**Table 1.** Characteristics of the study participants. Data are presented as mean (SD) unless otherwise stated.

	COPD_OB_	COPD_NW_	*p* value
*N*	80	80	
Age, years	64.5 (8.3)	65.1 (8.3)	0.63
Male, %	52.5	43.8	0.27
Pack years	36.6 (23.9)	36.8 (18.7)	0.97
Current smokers, %	23.8	36.2	0.21
Height, cm	170 (10)	168 (10)	0.29
Weight, kg	99.5 (15.4)	65.3 (7.7)	<0.01
BMI, kg/m^2^	34.5 (4.0)	23.0 (1.4)	<0.01
Waist circumference, cm	119 (10)	91 (8)	<0.01
Hip circumference, cm	111 (8)	96 (5)	<0.01
Waist/Hip ratio	1.08 (0.08)	0.95 (0.09)	<0.01
Fat, %	40.0 (8.8)	32.1 (6.7)	<0.01
FFMI, kg/m^2^	20.5 (2.5)	15.6 (1.5)	<0.01
FMI, kg/m^2^	13.9 (4.3)	7.4 (1.8)	<0.01
**Use of inhalers, %**			
SABA	38.8	48.8	0.20
SAMA	16.2	8.8	0.15
LABA	90.0	86.2	0.46
LAMA	82.5	86.2	0.66
ICS	67.5	65.0	0.74
**Known comorbidities, %**			
Diabetes mellitus	18.8	1.2	<0.01
Hypertension	45.0	27.5	0.02
Atrial fibrillation	2.5	3.8	0.65
Myocardial infarction	12.5	7.5	0.29
Heart failure	0.0	3.8	0.08
**SGRQ mean score**			
Symptoms	50.7	49.9	0.84
Impacts	32.2	28.8	0.25
Activity	64.7	57.7	0.06
Total	45.1	41.2	0.16

BMI: body mass index; FFMI: fat-free mass index; FMI: fat mass index; SABA: short-acting beta agonist; SAMA: short-acting muscarinic antagonist; LABA: long-acting beta agonist; LAMA: long-acting muscarinic antagonist; ICS: inhaled corticosteroid; SGRQ: St. George’s Respiratory Questionnaire.

### Pulmonary function and 6-minute walk test

Pulmonary function parameters and 6MWT results are presented in Table 2. By design, FEV_1_ was matched between COPD_OB_ and COPD_NW_ patients and averaged 1.47 L (SD 0.62) corresponding to 55.4 (SD 17.9) % predicted for the study population as a whole. While both groups were characterized by increased static lung volumes, COPD_OB_ patients showed significantly attenuated increases in TLC, FRC and RV compared to COPD_NW_ patients. Mean ERV % predicted was decreased by 24.6% (SD 3.8%) in the COPD_OB_ patients, while it was increased with 25.9% (SD 5.0%) in the COPD_NW_ patients (*p* < 0.01). The loss of diffusing capacity of the lungs for carbon monoxide (DL_CO_) was also attenuated in COPD_OB_ patients compared to COPD_NW_ patients.

**Table 2.** Pulmonary function and 6MWT. Data are presented as mean (SD) unless otherwise stated.

	COPD_OB_	COPD_NW_	*p* value
**Pulmonary function**			
FEV1, % predicted	56.2 (17.7)	54.6 (18.1)	0.57
FEV1, L	1.54 (0.65)	1.40 (0.58)	0.14
VC, % predicted	95.1 (17.4)	103.5 (23.0)	0.01
VC, L	3.36 (1.01)	3.41 (1.03)	0.73
FVC, % predicted	89.0 (18.0)	99.2 (21.2)	<0.01
FVC, L	3.06 (0.97)	3.17 (1.01)	0.49
TLC, % predicted	113.9 (16.6)	124.6 (17.6)	<0.01
TLC, L	6.82 (1.55)	7.18 (1.55)	0.15
FRC, % predicted	139.1 (30.5)	164.5 (33.0)	<0.01
FRC, L	4.42 (1.21)	5.08 (1.27)	<0.01
IC, % predicted	102.2 (23.4)	94.7 (24.0)	0.04
IC, L	2.64 (0.85)	2.29 (0.70)	<0.01
ERV,% predicted	75.4 (34.0)	125.9 (45.0)	<0.01
ERV, L	0.71 (0.39)	1.12 (0.52)	<0.01
RV, % predicted	155.6 (37.7)	175.1 (49.2)	<0.01
RV, L	3.46 (0.94)	3.81 (1.25)	0.04
RV/TLC ratio, %	51.2 (8.3)	52.9 (11.4)	0.28
FRC/TLC ratio, %	64.7 (8.1)	70.7 (7.5)	<0.01
DL_CO_ SB, % predicted	68.7 (17.8)	51.5 (15.1)	<0.01
DL_CO_/VA, % predicted	84.3 (22.1)	61.0 (19.1)	<0.01
**6MWT**			
6MWT, % predicted	81.8 (15.6)	76.6 (19.0)	0.06
6MWD, m	386 (80)	403 (103)	0.26
6MWW, kg/m	38542 (9535)	26508 (8141)	<0.01
SpO2 at rest,%	94 (3)	95 (3)	0.05
6MWT-induced drop in SpO2, %	4.4 (5.2)	4.7 (5.1)	0.67
**MPT**			
FRC increase upon MPT, %	20.2 (10.1)	19.5 (13.7)	0.71
delta FRC, L	0.51 (0.26)	0.42 (0.52)	0.09

FEV_1_: forced expiratory volume in 1 s; VC: vital capacity; FVC: forced vital capacity; TLC: total lung capacity; FRC: functional residual capacity; IC: inspiratory capacity; ERV: expiratory reserve volume; RV: residual volume; DL_CO_: diffusing capacity of the lungs for carbon monoxide; VA: alveolar volume; 6MWT: 6 min walk test; 6MWD: 6 min walking distance; 6MWW: walk work (6MWD x weight); SpO_2_: peripheral oxygen saturation; MPT: metronome paced tachypnea.

Remark: due to missing values DLCO: *n* = 78 in both COPD_OB_ and COPD_NW_; TLC, RV: *n* = 79 in COPD_NW_; 6MWT: *n* = 78 in COPD_OB_, *n* = 79 in COPD_NW_; MPT: *n* = 79 in COPD_OB_.

The 6MWD was comparable between both groups; however, the COPD_OB_ had significantly higher walk work (6MWW) as expected. While both groups showed a significant desaturation on average, the magnitude of desaturation was similar. Resting peripheral oxygen saturation was comparable between the groups. Finally, despite the observed differences in resting lung hyperinflation, both groups showed similar absolute levels of dynamic hyperinflation upon the MPT test.

### Dyspnea assessed by mMRC

**Figure 1.**
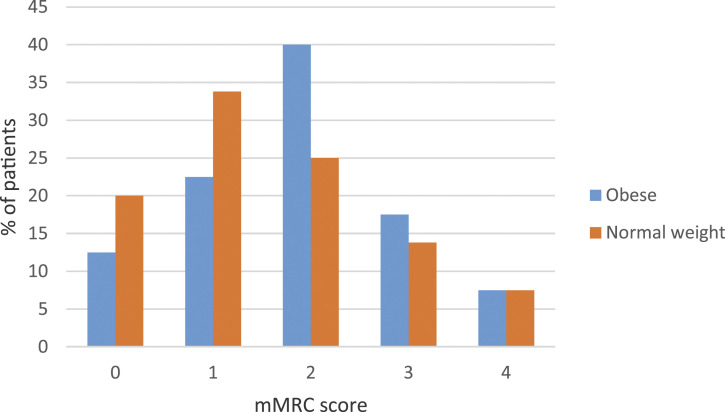
Modified medical research council dyspnea scale. Percentage of COPD_OB_ and COPD_NW_ with certain mMRC score. Abbreviations: mMRC, modified medical research council dyspnea scale.

mMRC dyspnea scores for COPD_OB_ and COPD_NW_ patients are presented in Figure 1. The proportion of patients with mMRC ≥ 2 was 65.0% in COPD_OB_ and 46.2% in COPD_NW_ (*p* = 0.02). The unadjusted OR of mMRC ≥ 2 was 2.2 (95% CI 1.1–4.1; *p* = 0.02) for COPD_OB_ compared to COPD_NW._ The adjusted logistic regression models for the association of BMI group with mMRC ≥ 2 are presented in Table 3. Addition of resting lung hyperinflation marker FRC to the model increased the OR of obesity for mMRC ≥ 2.

**Table 3.** Independent associations of BMI group (obese versus normal weight) with mMRC ≥ 2 and ΔBorg ≥ 2 during the 6MWT by different models of logistic regression. Model 1: adjusted for age, sex, smoking status, smoking pack years, known comorbidities and 6MWD % pred. (for ΔBorg ≥ 2 only). Model 2: as model 1 with additional adjustment for FRC, % pred. Model 3: as model 2 but with adjustment for 6MWW (walk work) instead of 6MWD % pred.

	Model 1		Model 2		Model 3	
**mMRC ≥ 2**	OR (95 %CI)	*p*	OR (95 %CI)	*p*	OR (95 %CI)	*p*
BMI group						
COPD_NW_	Reference	—	Reference	—	—	
COPD_OB_	3.0 (1.4–6.2)	<0.01	4.5 (2.0–10.2)	<0.01	—	
**ΔBorg ≥ 2**						
BMI group						
COPD_NW_	Reference	—	Reference	—	Reference	—
COPD_OB_	2.4 (1.1–5.2)	0.03	2.9 (1.3–6.7)	0.01	4.19 (1.6–10.7)	<0.01

FRC: functional residual capacity.

### Dyspnea by borg scores at 6MWT

Dyspnea was also measured at the start of 6MWT and at the end of the test with the Borg scale. The Borg dyspnea scores in COPD_OB_ group increased from 2.0 at rest to 4.0 at the end of 6MWT. In COPD_NW_, the Borg score increased from 1.8 at rest to 3.2 at the end of 6MWT. The ΔBorg upon 6MWT was significantly higher in COPD_OB_ than that of COPD_NW_ (2.0 vs 1.4; *p* = 0.01). In COPD_OB_, 39 (50%) patients had an increase of ≥ 2 in Borg score at the end of 6MWT, while in COPD_NW_, 27 (34%) showed such an increase (*p =* 0.04). Adjusted logistic regression models for the association between BMI group and ΔBorg scores during the 6MWT are presented in Table 3.

### Associations between measures of body composition and dyspnea

To provide more insight into the relation of body composition with mMRC (Supplement Table 1) and ΔBorg upon 6MWT (Supplement Table 2), we performed additional hypothesis-generating post hoc analyses. For this, we determined the sex-specific median values for each body composition parameter within the COPD_NW_ and COPD_OB_ groups separately, and subsequently divided COPD_NW_ and COPD_OB_ patients into “low” and “high” with respect to their sex- and group-specific medians. The COPD_NW_ “low” group was taken as the reference in logistic regression analyses. The results in Supplement Tables 1 and 2 show that whereas overall the COPD_OB_ low subgroups already had increased odds of having mMRC ≥ 2 and ΔBorg ≥ 2, these odds were even further increased considerably in the COPD_OB_ subgroups with the highest sex-specific BMI, WC, WHR, %fat, FFMI and FMI.

## Discussion

In this study, we compared pulmonary function and measures of weight-bearing exercise-induced dyspnea between FEV_1_- and age-matched obese and normal-weight COPD patients. COPD_OB_ patients had less resting lung hyperinflation, yet they reported higher mMRC and higher Borg scores at the end of the 6MWT. Furthermore, markers of (abdominal) adiposity appeared to significantly affect mMRC and ΔBorg upon 6MWT in our cohort of COPD patients.

Previous studies assessing dyspnea during weight-bearing exercise or using (m)MRC showed conflicting results. For example, a study by Rodríguez et al. indicated similar mMRC and Borg dyspnea scores at end of 6MWT between obese and normal-weight COPD groups.^21^ This is supported by other studies, where ΔBorg scores during 6MWT,^22^ Borg scores during domestic ADL’s like washing dishes and sweeping the floor^23^ and mMRC^24,25^ were comparable between obese and normal-weight COPD. To the contrary, others reported increased dyspnea in obese COPD patients.^22,26,27^ We designed our study by taking the confounders and limitations of these studies into account. As age and severity of airflow obstruction are determinants of symptom perception,^5^ we excluded the effects of these variables by matching the groups for age and FEV_1_. We did not include patients with comorbidities such as asthma, OSAS, recent acute cardiovascular event, atrial fibrillation at time of inclusion and lung diseases causing restriction such as interstitial lung diseases to minimize confounding.

Our results suggest that obesity in COPD is associated with increased weight-bearing exercise-induced dyspnea. Several factors seem to influence dyspnea ratings in obese COPD patients. Generally, lung hyperinflation is one of the determinants of increased dyspnea and poor QoL in COPD.^7,35,36^ In our study, the degree of dynamic lung hyperinflation, as measured with MPT, was comparable between COPD_OB_ and COPD_NW_ and thus was non relevant in the difference in dyspnea between the groups. However, the static lung volumes of COPD_OB_ were lower than COPD_NW_. This is in line with earlier reports, including comparable obese groups (mean BMI 32–35 kg/m^2^).^17–19^ Like these earlier studies, obesity was associated with lower FRC values with ERV being the most affected compartment. However, despite less resting lung hyperinflation, our COPD_OB_ patients had higher dyspnea ratings than their normal-weight counterparts. Our data suggest that this may at least partly be due to the negative effects of increased adiposity. Indeed, while COPD_OB_ patients have higher odds for mMRC ≥ 2 compared to COPD_NW_, these odds ratios are even greater when we adjust the models for resting lung hyperinflation. Our data suggest that the positive effects of obesity on pulmonary function protect obese patients to some degree of excess dyspnea. However, the positive effects of obesity in COPD, that is, less hyperinflation and better DL_CO_ apparently do not outweigh the negative effects of excess weight.

In concordance with the mMRC ratings, COPD_OB_ patients reported significantly higher increase in dyspnea during weight-bearing exertion. This is supported by earlier reports indicating that with obesity particularly weight-bearing exercise capacity is affected, probably due to increased work of breathing due to carrying excess weight.^37,38^ However, definite proof of this hypothesis is lacking and future studies with matched groups need to address this issue. The significantly higher walk work (6MWW) in COPD_OB_ appeared not to be a determinant of increased dyspnea during the 6MWT in our analyses. As with the mMRC, adjusting the models for resting lung hyperinflation resulted in increased odds for dyspnea during exertion, suggesting a protective role for obesity. Although the differences in dyspnea were statistically significant, the question remains whether these small differences (0.6 as measured with ΔBorg in disadvantage of obese) are clinically relevant. Either way, as also shown with adjusting the models for resting hyperinflation, the lower resting hyperinflation as a consequence of obesity seems to clearly protect obese COPD patients from increased dyspnea.

In this study, we also analyzed the role of different anthropometric and body composition measures on dyspnea. These analyses indicate that patients with central obesity are more likely to experience worse dyspnea as demonstrated by the strong association between increased WHR and dyspnea. Furthermore, the amount of fat as measured by FMI and fat % seems to be stronger associated with higher dyspnea ratings (both mMRC and ΔBorg during 6MWT) than BMI or FFMI. This suggests that primarily the amount of fat, and perhaps its location (central), are determinants of worse dyspnea with increasing weight in COPD patients. This finding, combined with accumulating data indicating abdominal adiposity as a risk factor for developing COPD,^39^ is important for future studies to develop treatment strategies specifically targeting subjects with central adiposity. Because FFMI was significantly higher in the obese group, we cannot fully rule out that the excess weight in this group might be due to a training effect and that this might had a beneficial effect on dyspnea and QoL. Future studies measuring muscle strength and cardiorespiratory fitness may provide more insight in this complex trade-off.

Our study has some limitations. This was a single center study conducted in a secondary care hospital with COPD patients who had on average moderate airflow limitation. Those with milder disease as well as those with very severe COPD may be underrepresented. Therefore our findings may not be generalizable to the whole COPD population. Furthermore, while our results indicate that obese COPD patients experience more weight-bearing exercise-induced dyspnea, we do not know whether this is associated with sedentary behavior since we did not perform accelerometry to objectively assess daily physical activity. It is plausible that patients who experience more weight-bearing exercise-induced dyspnea perform less of those activities in daily living.^40^ Also, we did not measure parameters of ventilation and gas exchange during exertion. Therefore, we could not compare or match for cardiorespiratory fitness. It should also be mentioned that this study was not designed to assess physiological mechanisms leading to dyspnea. Therefore, this study cannot provide in a thorough mechanistic explanation for the results. Future studies are needed in order to unravel these mechanisms.

In conclusion, our study indicates that despite its beneficial effect on resting lung hyperinflation, adiposity is associated with increased weight-bearing exercise-induced dyspnea in COPD.

## Supplemental Material

sj-pdf-1-crd-10.1177_14799731211052305 – Supplemental Material for Adiposity increases weight-bearing exercise-induced dyspnea despite favoring resting lung hyperinflation in COPD Click here for additional data file. Supplemental Material, sj-pdf-1-crd-10.1177_14799731211052305 for Adiposity increases weight-bearing exercise-induced dyspnea despite favoring resting lung hyperinflation in COPD by Zewari S, van den Borst B, van den Elshout FJ, Vercoulen JH, Dekhuijzen PN, Heijdra YF and Vos PJ in Chronic Respiratory Disease
