# Supplementary material for: Adiposity increases weight-bearing exercise-induced dyspnea despite favoring resting lung hyperinflation in COPD
Source: Chron Respir Dis. 2022 Feb 5;19:14799731211052305. doi: 10.1177/14799731211052305 (PMC8819751; doi:10.1177/14799731211052305)
Supplement: sj-pdf-1-crd-10.1177_14799731211052305 – Supplemental Material for Adiposity increases weight-bearing exercise-induced dyspnea despite favoring resting lung hyperinflation in COPD [file sj-pdf-1-crd-10.1177_14799731211052305.pdf]

Supplemental Tables

	Model 1		Model 2	
	OR (95% CI)	P	OR (95% CI)	P
BMI classes				
NW low	Reference		Reference	
NW high	1.16 (0.46 - 2.96)	0.75	1.80 (0.66 - 4.93)	0.26
OB low	2.21 (0.82 - 5.93)	0.12	4.28 (1.40 - 13.11)	0.01
OB high	3.96 (1.46 - 10.71)	<0.01	8.47 (2.26 - 27.33)	<0.01
WC classes				
NW low	Reference		Reference	
NW high	1.05 (0.41 - 2.68)	0.93	1.25 (0.47 - 3.36)	0.65
OB low	2.05 (0.77 - 5.43)	0.15	3.31 (1.15 - 9.54)	0.03
OB high	4.32 (1.48 - 12.68)	<0.01	8.16 (2.47 - 26.97)	<0.01
Waist-to-hip ratio classes				
NW low	Reference		Reference	
NW high	1.53 (0.59 - 3.96)	0.38	1.61 (0.61 - 4.49)	0.32
OB low	1.92 (0.72 - 5.11)	0.19	2.95 (1.03 - 8.45)	0.04
OB high	7.39 (2.43 - 22.53)	<0.01	12.81 (3.80 - 43.22)	<0.01
FFMI classes				
NW low	1.43 (0.55 - 3.68)	0.46	1.15 (0.43 - 3.07)	0.78
NW high	Reference		Reference	
OB low	2.98 (1.11 - 8.01)	0.03	4.15 (1.46 - 11.79)	<0.01
OB high	3.68 (1.35 - 10.09)	0.01	4.87 (1.69 - 14.00)	<0.01
FMI classes				
NW low	Reference		Reference	
NW high	1.97 (0.72 - 5.39)	0.19	2.62 (0.90 - 7.64)	0.08
OB low	2.37 (0.87 - 6.46)	0.09	4.20 (1.38 - 12.76)	0.01
OB high	7.02 (2.37 - 20.83)	<0.01	14.56 (4.19 - 50.58)	<0.01
Fat % classes				
NW low	Reference		Reference	
NW high	2.18 (0.77 - 6.14)	0.14	2.50 (0.84 - 7.38)	0.10
OB low	2.86 (1.04 - 7.91)	0.04	4.41 (1.47 - 13.23)	<0.01
OB high	6.57 (2.17 - 19.92)	<0.01	13.43 (3.80 - 47.48)	<0.01

Supplement Table 1. *Association between anthropometric and body composition measures and mMRC $\geq$ 2. For each variable the sex specific median for obese and normal weight groups were calculated. Obese subjects with values $\geq$ sex specific median were marked as 'OB high', while normal weight subjects were marked as 'NW high'. The OR of scoring mMRC $\geq$ 2 are presented for each variable. Model 1: adjusted for age, smoking status, smoking packyears, known comorbidities Model 2: as model 1 with additional adjustment for FRC, % pred.*

Abbreviations: *mMRC, modified medical research council dyspnea scale; BMI, body mass index; WC, waist circumference; FFMI, fat free mass index; FMI, fat mass index; FRC, functional residual capacity.*

	Model 1		Model 2	
	OR (95% CI)	P	OR (95% CI)	P
BMI classes				
NW low	Reference		Reference	
NW high	1.28 (0.49 - 3.35)	0.61	1.82 (0.65 - 5.06)	0.25
OB low	1.82 (0.67 - 4.94)	0.24	3.04 (1.01 - 9.16)	0.05
OB high	2.15 (0.83 - 5.59)	0.12	3.66 (1.24 - 10.80)	0.02
WC classes				
NW low	Reference		Reference	
NW high	1.14 (0.43 - 2.98)	0.80	1.35 (0.50 - 3.66)	0.55
OB low	2.05 (0.78 - 5.44)	0.15	2.98 (1.05 - 8.46)	0.04
OB high	1.70 (0.62 - 4.66)	0.30	2.62 (0.87 - 7.82)	0.09
Waist-to-hip ratio classes				
NW low	Reference		Reference	
NW high	1.45 (0.55 - 3.86)	0.46	1.63 (0.59 - 4.51)	0.35
OB low	1.22 (0.45 - 3.34)	0.70	1.74 (0.60 - 5.06)	0.31
OB high	3.80 (1.36 - 10.56)	0.01	5.87 (1.92 - 17.91)	<0.01
FFMI classes				
NW low	0.79 (0.30-2.10)	0.64	0.62 (0.22 - 1.72)	0.36
NW high	Reference		Reference	
OB low	1.69 (0.65 - 4.39)	0.28	2.18 (0.80 - 5.94)	0.13
OB high	1.44 (0.54 - 3.81)	0.46	1.69 (0.62 - 4.61)	0.31
FMI classes				
NW low	Reference		Reference	
NW high	3.97 (1.35 - 11.66)	0.01	4.87 (1.58 - 15.05)	<0.01
OB low	3.43 (1.18 - 10.00)	0.02	5.33 (1.68 - 16.96)	<0.01
OB high	4.27 (1.46 - 12.50)	<0.01	7.17 (2.17 - 23.76)	<0.01
Fat % classes				
NW low	Reference		Reference	
NW high	4.40 (1.44 - 13.43)	0.01	4.78 (1.51 - 15.11)	<0.01
OB low	3.26 (1.10 - 9.62)	0.03	4.40 (1.41 - 13.73)	0.01
OB high	5.21 (1.71 - 15.93)	<0.01	8.63 (2.48 - 30.03)	<0.01

Supplement Table 2. *Association between anthropometric and body composition measures and Δ Borg score during 6MWT. For each variable the sex specific median for obese and normal weight groups were calculated. Obese subjects with values ≥ sex specific median were marked as 'OB high', while normal weight subjects were marked as 'NW high'. The OR of Δ Borg score ≥ 2 during 6MWT are presented for each variable. Model 1: adjusted for age, smoking status, smoking packyears, known comorbidities Model 2: as model 1 with additional adjustment for FRC, % pred.*

Abbreviations: *mMRC, modified medical research council dyspnea scale; BMI, body mass index; WC, waist circumference; FFMI, fat free mass index; FMI, fat mass index; FRC, functional residual capacity.*
